# Memories of Lost Loved Ones

**DOI:** 10.1093/geroni/igaa057.2046

**Published:** 2020-12-16

**Authors:** Emily Mroz, Susan Bluck, Deborah Carr

## Abstract

The death of a loved one is a challenging but also normative occurrence in later life (e.g., Thomson et al, 2018). Experiencing the death of others typically increases with age, so personal reaction to loss becomes an ongoing process (Harrop et al., 2016). When adults lose someone, the deceased person is often ‘gone but not forgotten.’ That is, they are remembered over time (Klass & Steffen, 2017). The way one remembers their lost loved one’s life and their death (e.g., Mroz et al., 2019) may influence post-loss emotional adjustment and personal views. This symposium brings together Psychology and Sociology researchers with data from Germany, the US, and China whose work elucidates the complex relation between loss and memory: we identify how remembering lost loved ones relates to both adaptive and difficult outcomes. In this symposium, Wolf et al. examine beneficial and harmful ways of using autobiographical memories after a personal loss. Mroz and Bluck identify how grief responses in older adult widows lead to functional use of memories from the very end of the spouse’s life. Fu and Idler focus on the directive function of autobiographical memory, examining how memory for end-of-life experience with loved ones influences current choices for aggressive end-of-life care. Bolkan and Weaver examine how early life experiences with loss influence later personal views and advance care planning. Our Discussant, Debby Carr, integrates these talks to elucidate how remembering loss experiences relate to not only current grief, but also to people’s preparations for the future.

